# Elevated neopterin and decreased IL-4, BDNF levels and depression in lymphoma patients receiving R-CHOP chemotherapy

**DOI:** 10.3389/fneur.2024.1392275

**Published:** 2024-09-17

**Authors:** Pinki Mishra, Dinesh Bhurani, Nidhi

**Affiliations:** ^1^Department of Translational and Clinical Research, School of Chemical and Life Sciences, Jamia Hamdard, New Delhi, India; ^2^Department of Hemato-Oncology and Bone Marrow Transplant, Rajiv Gandhi Cancer Institute and Research Centre, Rohini, India; ^3^Department of Translational and Clinical Research, School of Chemical and Life Sciences, Jamia Hamdard, New Delhi, India

**Keywords:** depression, PHQ-9, BDNF, IL-4, neopterin, lymphoma, chemotherapy

## Abstract

**Objective:**

Depression is the most commonly observed psychological manifestation experienced by individuals diagnosed with cancer. The purpose of the study was to investigate the association between levels of IL-4, BDNF, neopterin, and depressive symptoms in lymphoma patients receiving consecutive cycles of chemotherapy.

**Methods:**

Newly diagnosed lymphoma patients scheduled to receive R-CHOP chemotherapy were enrolled. Effects of R-CHOP on circulatory biomarkers and depressive symptoms were assessed at three-time points [baseline assessment 7 days before the first dose of chemotherapy (TP1), interim assessment after the third cycle of chemotherapy (TP2), and follow-up assessment after the 6th cycle of chemotherapy (TP3)].

**Results:**

Seventy lymphoma patients, with a mean age of 44.17 ± 13.67 years, were enrolled. Patients receiving R-CHOP were found significantly increased neopterin levels between given time points TP1 vs. TP2, TP1 vs. TP3, and TP2 vs. TP3 (*p* < 0.001). However, IL-4 and BDNF levels significantly decreased with consecutive cycles of chemotherapy (*p* < 0.001). On Patient Health Questionnaire assessment (PHQ-9), scores of items like loss of interest, feeling depressed, sleep problems, loss of energy, and appetite problems were found significantly affected with consecutive cycles of chemotherapy (*p* < 0.001). The study found weak negative correlations between IL-4, BDNF, and neopterin levels and changes in PHQ-9 scores at both TP2 and TP3, suggesting a potential inverse relationship between these markers and depression symptoms.

**Conclusion:**

In conclusion, the present study suggests a potential link between elevated neopterin levels, decreased IL-4, and BDNF levels, and the presence of depression in lymphoma patients receiving R-CHOP chemotherapy. This study provides valuable insights into understanding the emotional challenges faced by cancer patients, offering information for more personalized interventions and comprehensive support approaches within the oncology setting.

## 1 Introduction

Lymphoma is a malignant neoplasm of immune cells that originates from B and T lymphocytes as well as natural killer cells. It includes a wide range of tumors with varying occurrence rates, etiology, pathogenesis, pathologic features, and clinical outcomes, and is commonly categorized as non-Hodgkin lymphoma (NHL) or Hodgkin lymphoma (HL) (1, 2). NHL was the 11th most frequently diagnosed cancer, with approximately 545,000 new cases, and the 11th leading contributor to cancer-related mortality in 2020, with an estimated 260,000 deaths (3). Rituximab, cyclophosphamide, doxorubicin, vincristine, and prednisone (R-CHOP) regimen is the existing standard of treatment for more than 60% of NHL patients (4). While advancements in treatment have improved survival rates, the impact of lymphoma extends beyond the real world, often influencing the psychosocial well-being of affected individuals (5). Numerous neuropsychiatric symptoms, such as depression, are present in cancer patients, and this condition is linked to a lower quality of life (6–8). About 25% of cancer patients experience depression related to their illness, and 6–13% of cancer patients cross-sectionally meet the diagnostic criteria for major depression (9). The interplay between these symptoms and biologic stressors, such as pain and the burden of physical symptoms, often impacts cancer progression and survival of the patients (10). Consequently, recognizing and effectively managing these disorders emerges as a crucial concern within the field of oncology practice (11, 12).

Depression is considered to be among the most commonly observed psychological manifestations experienced by individuals diagnosed with cancer (13). Its severity tends to increase during the administration of chemotherapy and persists for a significant duration following the completion of treatment. Moreover, it becomes evident in the reoccurrence of the disease and ultimately represents an independent prognostic determinant for mortality (14). Evidence indicates that biological mechanisms may be significant in addition to the evident emotional and psychosocial aspects of depression in cancer (15). Depression is recognized as a psychoneuroimmunological disorder, where a variety of behavioral, neuroendocrine, and neurochemical changes linked to these psychological disorders are believed to be caused by peripheral immune activation via the release of proinflammatory cytokines (16–19). Highlighting the potential role of the immune system in the onset of depression is likely to unveil new pathways in the field of psychopathology.

In addition to its robust correlation with depression, inflammation plays pivotal roles throughout all phases of tumor development (20). Within the tumor microenvironment, various cell types, including infiltrated inflammatory cells, endothelial cells, tumor-associated fibroblasts, and predominantly epithelial cells, including tumor cells, generate proinflammatory cytokines (21). While markers such as IL-6, TNF-alpha, and CRP have well-established relationships with depression in cancer patients (22), the present study focuses on IL-4, BDNF, and neopterin due to their unique and potentially significant roles in the inflammatory and neurotrophic pathways. IL-4 is a cytokine with known anti-inflammatory properties that might modulate immune responses differently from the pro-inflammatory markers like IL-6 and TNF-alpha (22, 23). In the context of cancer, the intricate interplay between the immune system and mental health is increasingly recognized. Studies suggest that IL-4 may play a role in regulating the immune response to cancer cells, but its influence on the psychological well-being of cancer patients, particularly in relation to depression, is complex (24). In previous studies, a negative relationship between psychological symptoms and IL-4 in cancer patients has been observed (19, 25). By investigating IL-4, we aim to understand the association between pro- and anti-inflammatory processes in depression associated with cancer. BDNF, a neurotrophin widely found in the central nervous system, is prominently distributed in regions such as the prefrontal cortex and hippocampus (26). BDNF has been involved in the pathophysiology of depression in cancer patients. Studies have shown that decreased BDNF levels are associated with cognitive impairment and depression in patients with cancer (27). BDNF plays a crucial role in synaptic activity and long-term synaptic memories, and changes in its levels can influence learning, memory, and depression-like behaviors (28). Additionally, BDNF has been found to be a potent protective factor against neurodegeneration, including in Alzheimer’s disease (29). Its involvement in depression is well-documented in other contexts, and its levels might provide insights into neurobiological changes specific to cancer patients undergoing chemotherapy. Therefore, BDNF and its association with depression in cancer patients is an important area of research that can contribute to the understanding and treatment of depression in this population.

Neopterin, a biomarker of immune system activation and oxidative stress, which is increasingly recognized as an important marker in the pathophysiology of depression. Elevated neopterin levels might reflect a distinct pathway linking immune activation to depressive symptoms in cancer patients (30). Wachter et al., first discovered high neopterin concentrations in the urine of cancer and viral infection patients (31). Following the initial discovery of elevated levels of neopterin in the urine of cancer patients (31), researchers have explored the presence of neopterin in the blood or urine of individuals with various types of cancer. Extensive research has been conducted on the levels of neopterin in the blood and urine of individuals diagnosed with gynecological tumors (32). Numerous investigations have recognized neopterin as a predictive indicator in gynecological cancers, such as cervical carcinoma (33) and ovarian cancer (34). Elevated levels of neopterin have been observed in individuals with hematological malignancies (35–37). In patients with lymphoma, increased concentrations of neopterin in urine have shown a correlation with response to therapy (38). Elevated neopterin levels have been documented not only following the administration of immune-activating agents such as cytokines but also after undergoing chemotherapy (39). Numerous studies have explored the blood levels of neopterin in patients with schizophrenia (SZ) and major depressive disorder (MDD) to date, revealing inconsistent findings. Studies have shown elevated neopterin plasma levels in individuals experiencing depression (40, 41).

There is paucity of data regarding mental health disorders among patients with hematologic malignancies (42–46), despite the fact that efforts to examine mental health disorders in oncology have focused primarily on solid malignancies. Although these molecules show promise as potential predictors of depression in cancer patients, it is important to note that the relationship between these biomarkers and depression is complex and multifaceted. However, the specific relationship between these biomarkers and depressive symptoms in the context of lymphoma remains relatively unexplored. This gap in knowledge is particularly pertinent given the unique challenges faced by individuals with lymphoma, including the impact of the disease itself, treatment-related side effects, and the psychological stress of living with a life-threatening illness. By focusing on these biomarkers, our study aims to uncover novel pathways and mechanisms that might be targeted for therapeutic interventions, thereby contributing to a more comprehensive understanding of depression in the oncology setting.

This study aims to address this gap by investigating the association between IL-4, BDNF, neopterin and depressive symptoms in lymphoma patients. Understanding the potential links between immune dysregulation and depressive symptomatology in this specific population holds implications for both clinical practice and theoretical frameworks that seek to elucidate the multifaceted connections between the immune system and mental health. Moreover, insights derived from this research may contribute to the development of targeted interventions aimed at mitigating depressive symptoms and improving the overall well-being of individuals navigating the complex landscape of lymphoma.

## 2 Methods

### 2.1 Study design and settings

This prospective longitudinal study recruited 70 newly diagnosed lymphoma patients between September 2020 and December 2021, following approval from the Institutional Review Board (IRB) of Rajiv Gandhi Cancer Institute and Research Centre, New Delhi, India. The aim was to study the impact of chemotherapy on depressive symptoms and its correlation with IL-4, BDNF, neopterin. The study focused on patients who were undergoing R-CHOP chemotherapy (comprising 375 mg/m^2^ rituximab, 750 mg/m^2^ cyclophosphamide; 50 mg/m^2^ doxorubicin; 1.4 mg/m^2^ vincristine, up to maximal dose of 2 mg on day 1; 40 mg/m^2^ prednisolone) as part of their treatment regimen. Written informed consent was obtained from every eligible patient, and strict measures were implemented to safeguard patient confidentiality.

### 2.2 Participants

The study recruited patients from a tertiary care hospital, focusing on individuals who met specific eligibility criteria. Inclusion criteria comprised newly diagnosed lymphoma patients confirmed through histological and cytological assessments, scheduled to undergo R-CHOP treatment, chemotherapy-naïve, and aged between 18 and 65 years. Exclusion criteria encompassed individuals below 18 years, patient with prior history of depression, a history of schizophrenia or other neuropsychiatric disorders, substance abuse, use of anti-inflammatory drugs, incompetence for interview, and lack of willingness to participate and provide consent for the study.

### 2.3 Assessment of depression using PHQ-9 in cancer patients

The Patient Health Questionnaire (PHQ-9), validated, self-report screening tool to measure the severity of depressive symptoms. Distinguishing itself from other depression scales, the PHQ-9 comprises nine items aligned with the Diagnostic and Statistical Manual of Mental Disorders, 5th edition (DSM-V) criteria for MDD (47). The questionnaire evaluates the frequency with which the participants were disturbed by any of the nine items in the 2 weeks prior. Each item on the PHQ-9 is rated on a scale of 0 to 3 (0 = not at all; 1 = several days; 2 = more than a week; 3 = nearly every day). The total PHQ-9 score ranges from 0 to 27, with scores of 5–9 categorized as mild depression, 10–14 as moderate depression, 15–19 as moderately severe depression, and ≥ 20 as severe depression (48). While the PHQ-9 total score provides a comprehensive assessment of depression severity, its single-score nature may obscure the differential impact of specific symptoms. By analyzing individual PHQ-9 items, we sought to explore potential differential relationships between specific symptoms (e.g., anhedonia, fatigue, concentration difficulties) and biomarkers in lymphoma patients undergoing chemotherapy. This approach allows us to identify potential symptom-specific effects that may not be apparent when considering the overall depression score.

### 2.4 Evaluation of IL-4, BDNF and neopterin levels in cancer patients

Before the initiation of each chemotherapy cycle, 5-mL of blood sample was withdrawn at each time-point (TP1, TP2 and TP3). The samples underwent centrifugation for 15 min at 4000 rpm to facilitate serum separation. The resulting supernatant serum was then transferred into two separate aliquots and stored at −80°C until analysis. Serum interleukin IL-4, BDNF and neopterin levels were quantified for each 50 μL of serum sample using a highly sensitive ELISA kits from Bioassay Technology Laboratory (Shanghai Korain Biotech Co., Ltd., Shanghai, China).

### 2.5 Statistical analysis

The statistical analysis was conducted using IBM SPSS software (version 25.0, IBM Corp., Armonk, NY, USA). Categorical variables were defined in terms of frequencies and corresponding proportions, while continuous variables were expressed as mean ± SD/Median (IQR). The Kolmogorov–Smirnov test was employed to assess the distribution of the collected data. Given the non-normal distribution, non-parametric tests were applied. Demographic data is presented based on the distribution of the respective data. Friedman’s Two-way Analysis of Variance by Ranks test was employed to analyze the impact of treatment modality and time interval on factors such as IL-4, BDNF, neopterin and PHQ-9 score in lymphoma patients, adjusting for covariates such as age, gender, and level of education to control for potential confounding effects of these demographic variables. Post-hoc analysis was done using Kruskal Wallis One-way ANOVA test. Statistical significance was set at a *p*-value less than 0.05, and the significance level was adjusted for multiple comparisons using Bonferroni’s correction to reduce the risk of Type I error. Associations that remained robust after Bonferroni adjustment for three comparisons (alpha = 0.0017) were duly noted. Correlation analyses were carried out using Spearman’s rank correlation coefficient rho (ρ). Percentage change was computed using the formula [% change = (Final value − Initial value) × 100/Initial value], with the median value at TP1 considered as the initial value and the median value at TP3 as the final value, to provide a clear measure of change over the treatment period. A post-hoc power analysis was conducted to justify the adequacy of the sample size, using G*Power software (version 3.1.9.4). We based our calculations on the following parameters: effect Size (f) = 0.403 calculated based on partial eta squared (*η*^2^) = 0.14; type-I error (*α*) = 0.05; power (1 − β): Targeted at 0.80, which is a commonly accepted threshold in clinical research.

## 3 Results

The present study included 70 lymphoma patients, 62.85% of them were male (44) and 37.15% were females (26), with a mean age of 44.17 ± 13.67 years. All the patients received R-CHOP as the main chemotherapy regimen. Table 1 displays the clinical and demographic features of the study participants.

**Table 1:** Baseline demographics of patients.

Factor	Lymphoma patients (*n* = 70)
Age (Mean ± SD) years	44.17 ± 13.67
Sex	
Male	44 (62.85)
Female	26 (37.15)
Performance status	
0	20 (28.57)
1	42 (60)
>1	8 (11.43)
B symptoms	
Present	23 (32.85)
Absent	41 (58.57)
Not known	6 (8.57)
Histological subtypes	
Diffuse large B-cell lymphoma	70 (100)
Stage	
I	4 (5.71)
II	13 (18.57)
III	9 (12.85)
IV	36 (51.42)
Not available	2 (2.85)
Immunophenotype	
GC	35 (50)
Non-GC	35 (50)
IPI score	
0	9 (12.85)
1	12 (17.14)
2	21 (29.17)
3	17 (24.28)
4	5 (7.14)
5	6 (8.57)
Education	
<High school	16 (22.85)
High school	34 (48.57)
Partial college and higher	20 (28.57)
Comorbidities	
Yes	30 (42.85)
No	40 (57.15)

Values are expressed as mean ± SD for continuous variables and number (%) for categorical variables.

IPI, International Prognostic Index. Immunophenotype: Non-GC type positive for CD20, Bcl-2 and MUM-1; GC type positive for CD20, CD10 and Bcl-6.

### 3.1 Effect of R-CHOP on IL-4, BDNF and neopterin levels in lymphoma patients

Patients who received R-CHOP chemotherapy were found with significantly decreased levels of IL-4 and BDNF between given time-points TP1 vs. TP2, TP1 vs. TP3 and TP2 vs. TP3 (*p* < 0.001). However, levels of neopterin were significantly elevated with consecutive cycles of chemotherapy (*p* < 0.001). For, BDNF and neopterin, the changes in the levels for TP2 to TP3 were not significant as shown in Table 2.

**Table 2:** The effect of different cycles of R-CHOP on IL-4, BDNF and neopterin levels of all lymphoma patients (*n* = 70).

Cytokine		Concentration			%age change TP1 to TP3	*p*-value	Post-hoc^a^ *p*-value		
		TP1	TP2	TP3			TP1 to TP2	TP1 to TP3	TP2 to TP3
IL-4 (pg/mL)	Mean (SD) Median (IQR)	81.98 (42.05) 75.13 (50.35, 103.23)	39 (23.01) 36.91 (19.18, 51.5)	23.04 (12.4) 24.39 (12.55, 31.42)	−71.89	<0.001^**^	<0.001^**^	<0.001^**^	0.001^**^
BDNF (ng/mL)	Mean (SD) Median (IQR)	39.36 (20.01) 41.43 (22.71, 53.75)	25.39 (15.41) 24.59 (13.52, 37.38)	19.35 (11.76) 18.03 (10.72, 26.87)	−50.83	<0.001^**^	<0.001^**^	<0.001^**^	0.088
Neopterin (ng/mL)	Mean (SD) Median (IQR)	9.61 (5.8) 8.71 (5.6, 13.48)	13.52 (8.47) 13.92 (8.06, 18.71)	17.23 (12.8) 15.05 (8.53, 25.88)	79.29	<0.001^**^	0.009^**^	<0.001^**^	0.790

*p < 0.05 statistically significant. **p < 0.017 Bonferroni adjusted threshold^a^.

Significance values have been adjusted by the Bonferroni correction for multiple tests.

Values are expressed as mean (SD)/median (IQR) for continuous variables.

### 3.2 Effect of R-CHOP on PHQ-9 scale

Scores of PHQ-9 items like Loss of interest (*p* < 0.001), feeling depressed (*p* < 0.001), sleep problems (*p* < 0.001), loss of energy (*p* < 0.001), and appetite problems (*p* < 0.001) were found significantly affected with consecutive cycles of chemotherapy (Table 3). The scores recorded at TP3 were significantly increased from the baseline scores. However, no change from TP1 to TP3 was observed in the scores of items like self-blame, concentration problem, agitation/retardation and suicidal ideation. The overall change in the total PHQ-9 score from TP1 to TP2 (*p* = 0.005), TP1 to TP3 (*p* < 0.001) and TP2 to TP3 (<0.001) were found significantly affected.

**Table 3:** Effects of R-CHOP (*n* = 70) on different items of PHQ-9 in lymphoma patients.

Item		PHQ-9 score			*p*-value	Post-hoc^a^ *p*-value		
		TP1	TP2	TP3		TP1 to TP2	TP1 to TP3	TP2 to TP3
Loss of interest	Mean (SD) Median (IQR)	0.54 (0.65) 0 (0,1)	0.86 (0.7) 1 (0,1)	1.4 (0.87) 1 (1,2)	<0.001^**^	0.043^*^	<0.001^**^	0.001^**^
Feeling depressed	Mean (SD) Median (IQR)	0.06 (0.23) 0 (0,0)	0.16 (0.5) 0 (0,0)	1.27 (0.88) 1 (1,2)	<0.001^**^	1.000	<0.001^**^	<0.001^**^
Sleep problems	Mean (SD) Median (IQR)	0.37 (0.54) 0 (0,1)	0.59 (0.78) 0 (0,1)	1.14 (0.98) 1 (0,2)	<0.001^**^	0.585	<0.001^**^	0.001^**^
Loss of energy	Mean (SD) Median (IQR)	0.23 (0.42) 0 (0,0)	0.43 (0.57) 0 (0,1)	0.64 (0.68) 1 (0,1)	<0.001^**^	0.143	<0.001^**^	0.154
Appetite problems	Mean (SD) Median (IQR)	0.21 (0.41) 0 (0,0)	0.41 (0.67) 0 (0,1)	0.63 (0.74) 0 (0,1)	0.001^**^	0.372	<0.001^**^	0.135
Self-blame	Mean (SD) Median (IQR)	0.07 (0.26) 0 (0,0)	0.1 (0.34) 0 (0,0)	0.14 (0.42) 0 (0,0)	0.406	ns	ns	ns
Concentration problems	Mean (SD) Median (IQR)	0.09 (0.28) 0 (0,0)	0.2 (0.43) 0 (0,0)	0.26 (0.5) 0 (0,0)	0.087	ns	ns	ns
Agitation/retardation	Mean (SD) Median (IQR)	0.06 (0.23) 0 (0,0)	0.17 (0.97) 0 (0,0)	0.19 (0.99) 0 (0,0)	0.641	ns	ns	ns
Suicidal ideation	Mean (SD) Median (IQR)	0.09 (0.28) 0 (0,0)	0.06 (0.28) 0 (0,0)	0.11 (0.62) 0 (0,0)	0.682	ns	ns	ns
Sum score	Mean (SD) Median (IQR)	1.71 (1.21) 1 (2,3)	2.97 (2.3) 1 (3,4)	5.79 (2.65) 4 (6,7)	<0.001^**^	0.005	<0.001	<0.001

*p < 0.05 statistically significant, **p < 0.017 Bonferroni adjusted threshold^a^.

Significance values have been adjusted by the Bonferroni correction for multiple tests.

Values are expressed as mean (SD)/median (IQR) for continuous variables.

### 3.3 Correlation coefficient for IL-4, BDNF and neopterin levels and PHQ-9 items

**Figure 1:**
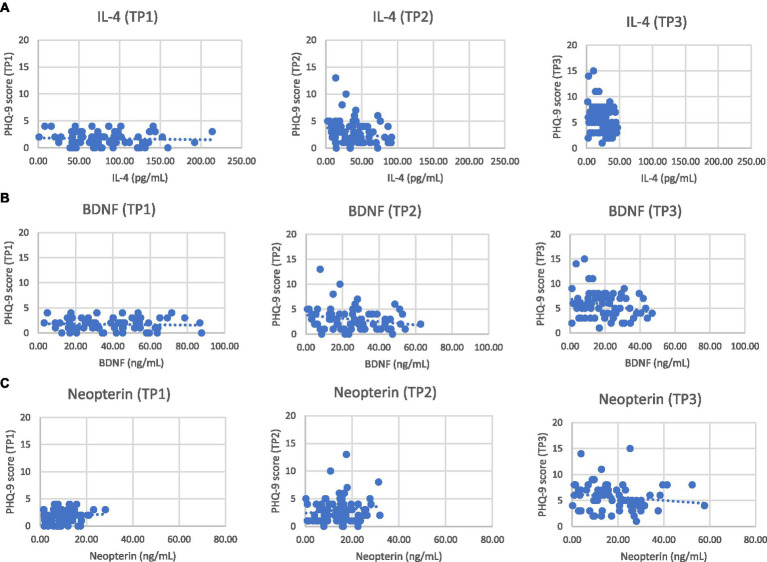
Scatter plots between PHQ-9 scores and serum levels of IL-4 **(A)** or BDNF **(B)** and neopterin **(C)** in NHL patients at different time points.

The correlation of IL-4, BDNF, and neopterin levels with the PHQ-9 score changes was assessed at TP2 and TP3 as shown in Figure 1. The results indicate weak negative associations for all markers with the changes in PHQ-9 scores at both time points, though none of these correlations were statistically significant. IL-4 shows a weak negative correlation with changes in PHQ-9 scores at both TP2 (*ρ* = −0.209) and TP3 (*ρ* = −0.223). A negative correlation indicates that as IL-4 levels increase, the PHQ-9 score decreases slightly, suggesting a potential inverse relationship between IL-4 and depression symptoms. However, the associations were not significant (*p*-values >0.05). BDNF also exhibits a weak negative correlation with changes in PHQ-9 scores at TP2 (*ρ* = −0.225) and TP3 (*ρ* = −0.212). Similar to IL-4, the negative correlation suggests that higher BDNF levels might be associated with lower PHQ-9 scores. The lack of statistical significance (*p*-values >0.05). Neopterin shows a weak negative correlation with changes in PHQ-9 scores at TP2 (*ρ* = −0.225) and TP3 (*ρ* = −0.203). Despite the weak negative correlation, an increase in neopterin levels with an increase in PHQ-9 scores was noted, which suggests a contradiction, as the weak negative correlation implies higher neopterin levels might generally be associated with lower PHQ-9 scores.

## 4 Discussion

In the present study, we investigated association between levels of IL-4, BDNF, neopterin and depression in lymphoma patients receiving consecutive cycles of chemotherapy. We observed significant alterations in the levels of these immune response markers throughout the treatment. Patients evaluated before chemotherapy presented higher concentrations of IL-4 and BDNF, which decreased following the cycles of chemotherapy. Conversely, low baseline neopterin levels increased post-chemotherapy treatment. A weak negative association of IL-4, BDNF and neopterin was observed with PHQ-9 scores. Additionally, significantly poorer performance in PHQ-9 items like loss of interest, feeling depressed, sleep problems, loss of energy, and appetite problems was found after consecutive cycles of chemotherapy.

The PHQ-9 is extensively employed in primary care settings to screen for clinical depression (48, 49). However, its application in clinical oncology groups has been limited (50–52). In clinical oncology research, the emphasis has primarily been on evaluating the PHQ-9’s diagnostic precision in identifying MDD, rather than profiling a group of cancer patients exhibiting depressive symptoms identified by the PHQ-9. A cross-sectional study conducted on cancer patients undergoing initial cycles of chemotherapy, both at day 0 and 28 days into the treatment, revealed an increase in PHQ-9 scores following the chemotherapy cycles (53). Similar findings have been observed in the present study, where we found an increase in the PHQ-9 item scores after receiving cycles of chemotherapy. Notable changes were observed in several PHQ-9 items in the present study, specifically in items like loss of interest, feeling depressed, sleep problems, loss of energy, and appetite problems (*p* < 0.001). These items, reflecting core symptoms of depression, were significantly affected by the consecutive cycles of R-CHOP chemotherapy. These findings can be explained by the facts that prolonged treatment durations, repeated admissions to the hospital, and the adverse effects of chemotherapy significantly affect patients’ mental and emotional health. This is likely due to the prolonged chemotherapy and repeated hospital visits negatively impacting their psychological health. Subsequent studies have reported deterioration in the psychological condition and occurrence of depression following cycles of chemotherapy in cancer patients (53, 54). The worsening of specific depressive symptoms is consistent with existing literature on inflammation-mediated depression. Inflammatory cytokines such as IL-6 and TNF-alpha are closely linked to anhedonia and depressed mood by disrupting the brain’s reward pathways (55). Elevated levels of IL-6 and CRP have been associated with sleep disturbances, as inflammation impacts the sleep–wake cycle and reduces sleep quality (55). Fatigue and loss of energy, common features of inflammatory depression, are driven by cytokine-induced sickness behavior, leading to lethargy and decreased motivation (56). Additionally, cytokines can affect hypothalamic function, contributing to changes in appetite, either increased or decreased (57). However, no changes were observed in items related to self-blame, concentration problems, agitation/retardation, and suicidal ideation. This might be due to the multifactorial nature of these symptoms, which are influenced by various biological, psychological, and social factors beyond inflammation alone. These symptoms may not be as directly linked to inflammatory pathways as the core symptoms of depression observed in our study. The observations highlight the critical role of inflammation in shaping various depressive symptoms, reinforcing the need to consider inflammatory pathways in the diagnosis and treatment of depression. Understanding these mechanisms may guide more targeted therapeutic approaches for managing depression, particularly in populations with increased inflammation. The PHQ-9 scores in the present study were comparatively low compared to those reported in similar studies involving cancer patients. Previous research has demonstrated varying rates of depression among cancer patients, with some studies indicating higher average PHQ-9 scores. For example, Walker et al. (58) found that 13% of cancer patients met the criteria for major depression based on the PHQ-9, with mean scores significantly exceeding those observed in the present study. Similarly, studies on patients undergoing chemotherapy frequently report higher PHQ-9 scores. Hartung et al. (51), for instance, recorded mean PHQ-9 scores ranging from moderate to severe depression levels in chemotherapy patients. The lower scores in our sample might be due to differences in patient demographics, disease stages, and the specific chemotherapy treatment administered.

Extensive evidence substantiates the bidirectional communication between the immune system and the central nervous system, often referred as “neuroimmune axis” (59, 60). The “cytokine hypothesis” of depression etiology suggests that proinflammatory cytokines produced by cells in the tumor microenvironment cause behavioral changes observed in cancer patients, which primarily affect the central nervous system function (57). Cytokines bind to specific receptors on nerve cells and can influence serotonin metabolism by altering the metabolism of tryptophan impacting the hypothalamic–pituitary–adrenal axis, leading to an increased inflammatory response that interrupts the functioning of glucocorticoid receptors. These cytokines may also modify neural plasticity, affecting mood regulation and contributing to depressive symptoms (12, 16).

IL-4 is a cytokine involved in regulating the immune system, mainly secreted by mast cells, Th2 cells, eosinophils and basophils (23). It stimulates the proliferation of activated B-cells and T-cells, as well as the differentiation of B-cells into plasma cells, playing a crucial role in the regulation of both the humoral and adaptive immune system. IL-4 has anti-inflammatory properties and can inhibit the production of Th1 cells, macrophages, and interferon-gamma (IFN-γ) (61). Research indicates that a person suffering from depression triggers the compensatory immunoregulatory response system (CIRS). This activation is a countermeasure against their hyperactive inflammatory response system (IRS), which helps maintain the immunological balance (62). Depressed patients exhibit elevated levels of pro-inflammatory cytokines, including IL-6, IL-1, TNF-α. To counterbalance the exaggerated inflammatory response induced by these pro-inflammatory cytokines, IL-4 is produced in large quantities. These anti-inflammatory cytokines perform their regulatory role by either inhibiting the production of pro-inflammatory cytokines or by promoting the development of phenotypes linked to M2 macrophages (63). The evidence supporting the link between IL-4 and depression is conflicting. Studies have shown elevated IL-4 levels in patients with severe depression (61, 64) while, others have demonstrated reduced IL-4 levels in acute depression (65, 66). A cohort study done in in early-stage breast cancer patients investigated the changes in cytokine (IL-1β, TNF-α and IL-4) levels before and after chemotherapy demonstrated higher IL-4 levels in patients after receiving the chemotherapy treatment (67). Furthermore, a notable reduction in the levels of IL-4 has been observed in aggressive NHL patients following the chemotherapy treatment (68). The present study revealed a significantly decreased IL-4 levels following cycles of chemotherapy and a negative correlation with PHQ-9 scores align with studies suggesting that major depression in cancer patients may be linked to inadequately controlled inflammation related to the disease.

Brain-derived neurotrophic factor (BDNF) is a protein primarily secreted by neurons within the brain, distributed across various regions (69). Notably, the hippocampus exhibits the highest concentrations of BDNF. This protein plays a crucial role in promoting neurogenesis and influencing neuroplasticity in the brain. BDNF can cross the BBB contributing to detectable levels in the blood (70). There is evidence to suggest that the pathophysiology of MDD is associated with low serum concentrations of BDNF (71, 72). Lower BDNF levels have been linked with chemotherapy-related cognitive impairment (73, 74) but the association with depression in cancer patients remains unclear (73, 75). According to preclinical studies, neurogenesis and proliferating neural stem cells in the hippocampus are markedly reduced when exposed to cytotoxic agents (76, 77). This chemotherapy-induced decrease in neurogenic and oligogenic populations in the brain was associated with reduced BDNF and TrkB activity in the hippocampus (78, 79). The decrease in BDNF levels may possibly be the consequence of cytokines inhibiting BDNF expression in the brain. Extensive studies on BDNF lend support to the “neurotrophic hypothesis” of depression. This hypothesis emphasizes that a reduction in BDNF levels is central to the onset of depression, leading to neuroplastic changes. These changes include the loss of neurons, diminished neurogenesis in the hippocampus, and a reduction in glial cells, all contributing to depressive symptoms (80).

In addition to the general role of BDNF in depression, genetic variations such as the Val66Met polymorphism have been identified as potential vulnerability factors, particularly in the context of inflammation. The Val66Met polymorphism, which involves the substitution of valine (Val) with methionine (Met) at codon 66, affects BDNF secretion and has been linked to various neuropsychiatric conditions, including depression (81). A cohort study demonstrated that women with the Met allele exhibited greater depressive symptoms in the presence of elevated inflammation compared to those with the Val/Val genotype, suggesting increased susceptibility to depression. This interaction between BDNF genotype and inflammation may explain why some cancer patients, particularly those undergoing chemotherapy, are more prone to depression (55). Although the present study did not assess BDNF genotypes, a significant decline in BDNF levels post-R-CHOP chemotherapy and an inverse relationship with PHQ-9 scores was noted suggesting the pathophysiological role of BDNF in chemotherapy-related depression. Future research incorporating genetic factors like the Val66Met polymorphism could refine our understanding and management of depression in lymphoma patients.

Neopterin is an aromatic pteridine produced by monocytes and macrophages following stimulation by IFN-γ, released by activated T-cells. It is proposed that neopterin serves as an indicative marker for the activation of the immune system. It plays a role in the growth, differentiation, and progression of tumors as well as the activation of oncogenes (82). Elevated neopterin levels have been observed in individuals with depression (40, 83). Furthermore, a meta-analysis of case–control studies revealed higher blood neopterin concentrations in individuals with depression compared to healthy controls (84). These findings show an association between neopterin levels and depression. Elevated neopterin levels have been observed in various conditions linked to immune activation. Research has shown that neopterin levels increase in various disorders associated with immune system activation, including cancer (30). Elevated neopterin levels have been observed after chemotherapy administration (39). Neopterin released by immune cells or adipose tissue can penetrate the CNS, triggering local immune activation. Activated immune cells such as monocytes, macrophages, and T cells can migrate from the periphery to the brain, where they produce cytokines. Chronic immune activation can cause microglia to release inflammatory mediators that affect neurotransmitter systems and neuronal health. Inflammatory cytokines are known to impact neuronal development, apoptosis, and brain-derived neurotrophic factor (BDNF) receptor (TrkB) phosphorylation, disrupting BDNF signaling. Neopterin released by peripheral immune cells or adipose tissue can access the CNS to initiate local immune activation, and activated immune cells such as monocytes/macrophages and T cells can be recruited from the periphery to the brain parenchyma, and these cells can in turn produce cytokines in the brain. Under conditions of chronic immune activation, microglia can become a source of inflammatory mediators that may influence brain neurotransmitter systems and neuronal integrity (85). The present study also demonstrated elevated neopterin levels following consecutive cycles of chemotherapy, Along with a weak correlation between neopterin levels and PHQ-9 scores. The strength of the present study was that it evaluated inflammation and depression in patients undergoing chemotherapy for up to six cycles, using a design specifically intended to evaluate these outcomes in patients with lymphoma. Measuring cytokines before and after each chemotherapy cycle helped to strengthen the study by revealing changes in the patients over time.

The present study has several limitations. First, the relatively small sample size and single-center design, which may limit the generalizability of the study findings. Although the post-hoc power analysis suggests that the sample size of 70 patients is sufficient to detect medium effect sizes, smaller effect sizes may not be identified with the same level of confidence. Future studies with larger sample size and multi-center cohorts could provide more robust and generalizable data. It was difficult to ascertain the connection between changes in the immune response markers, depression and the progression of cancer since the study did not include an NHL control group. In order to draw better comparisons, it could be more beneficial for future research to use both healthy and NHL controls who are not undergoing chemotherapy, as references. Including both kinds of controls aids in determining how chemotherapy and cancer affect depressive symptoms and the levels of immune markers. Despite the limitations of this study, we believe it has important clinical and research implications. Targeting biomarkers such as neopterin, IL-4, and BDNF may help in the management of depression in lymphoma patients undergoing chemotherapy. Neopterin levels can be reduced through anti-inflammatory therapies and immunomodulatory agents, potentially alleviating depressive symptoms. IL-4 modulation via regular physical activity, dietary interventions, and cytokine therapy may promote an anti-inflammatory state, contributing to improved mood and immune function. BDNF levels can be elevated through the use of antidepressants, structured exercise regimens, psychotherapy, and nutritional supplements like vitamin D, and omega-3 fatty acids, which support neuroplasticity and mitigate depression. These biomarker-focused interventions present a promising approach to personalized depression management, warranting further clinical validation.

## 5 Conclusion

In conclusion, our study provides valuable insights into the potential association between IL-4, BDNF, neopterin, and depressive symptoms in lymphoma patients undergoing R-CHOP chemotherapy. The results imply that these biomarkers might be involved in the mechanisms underlying depression in lymphoma patients undergoing R-CHOP chemotherapy. This has important implications for the management of psychological well-being in this patient population. Further research is warranted to elucidate the underlying mechanisms and to explore the potential of these biomarkers as therapeutic targets or prognostic indicators. Our work contributes to the growing body of evidence on the complex relationship between biological factors and depressive symptoms in cancer patients, and highlights the need for comprehensive support strategies in the oncology setting.

## Data Availability

The raw data supporting the conclusions of this article will be made available by the authors, without undue reservation.
